# Preoperative quality of life as prediction for severe postoperative complications in gynecological cancer surgery: results of a prospective study

**DOI:** 10.1007/s00404-020-05847-1

**Published:** 2020-10-29

**Authors:** Jalid Sehouli, Kathrin Heise, Rolf Richter, Hannah Woopen, Louise Anders, Melisa Guelhan Inci

**Affiliations:** Department of Gynecology with Center of Surgical Oncology, Charité University Hospital of Berlin, Augustenburger Platz 1, 13353 Berlin, Germany

**Keywords:** Quality of life (QoL), Oncology, Gynecology, Surgery, Postoperative complications

## Abstract

**Purpose:**

The aim of this study was to investigate preoperative quality of life (QoL) as a predictive tool for severe postoperative complications (POC) in gynecological cancer surgery.

**Methods:**

This is a prospective study of patients undergoing gynecologic cancer surgery at an academic center in Germany. QoL was assessed by the EORTC Quality of Life Questionnaire (QLQ-C30) and the NCCN Distress Thermometer (DT). Several geriatric assessment tools have been applied. POC were graded using Clavien–Dindo criteria. Using multivariable logistic regression models, we identified predictive clinical characteristics for postoperative complications.

**Results:**

Within 30 days of surgery, 40 patients (18%) experienced grade ≥ 3b complications including 9 patients (4%) who died. The dominant complication was anastomosis insufficiency with 13 patients (5.8%). In the multivariable stepwise logistic regression through all univariate significant variables, we found that impaired physical functioning was predictive of POC, defined by an EORTC score < 70 (OR 5.08, 95% CI 2.23–11.59, *p* < 0.001). Regarding symptoms nausea/vomiting assessed as an EORTC score > 20 (OR 3.08, 95% CI 1.15–8.26, *p* = 0.025) indicated a significant predictive value. Being overweight or obese (BMI > 25) were also identified as predictive factors (OR 5.44, 95% CI 2.04–14.49, *p* = 0.001) as were reduced Mini Mental State Examination (MMSE) results < 27 (OR 7.94, 95% CI 1.36–45.46, *p* = 0.02).

**Conclusion:**

Preoperative QoL measurements could help to predict postoperative complications in patients with gynecological cancer. Patients with limitations of mobility, debilitating symptoms and cognitive impairment have an increased risk for developing severe POC.

## Introduction

Surgery for advanced gynecological malignancies often requires complex and multivisceral procedures and can be associated with severe postoperative complications. The preoperative identification of predictive factors for postoperative complications (POC) may help to reduce patient’s morbidity and mortality.

So far there is no standardized routine for reliably predicting a patient’s risk of POC [1]. Many of the commonly used instruments and measures such as age, Eastern Cooperative Oncology Group (ECOG) performance status, the American Society of Anesthesiologists (ASA) physical status classification system or comorbidities are not sufficient because they lack sensitivity and do not assess physiological reserve [2, 3].

The assessment of a patient’s QoL is well established in clinical oncology to describe the side effects of cancer therapies. QoL has previously been recognized as an independent predictor of survival and has a significant prognostic value in cancer patients [4–7]. A patient’s quality of life is impacted by numerous aspects of their life such as physical status, social functioning as well as symptom burden and distress levels [8, 9]. Especially functioning domains such as low physical functioning and some specific symptoms (most notably pain, fatigue and loss of appetite) have been associated with poorer survival in cancer patients [5, 10]. Nevertheless, systematic QoL measurements are not regularly applied in surgery. In this context, we performed this prospective trial to explore the role of a multidimensional QoL measurement to predict severe postoperative complications.

## Materials and methods

### Study design

This paper is part of a project concerning the role of predictive markers for severe POC in Gynecological Cancer Surgery: The RISC-Gyn trial.

Data were collected in a prospective non-interventional cohort study including 237 women who underwent elective surgery for a gynecologic malignancy.

Recruitment started in October 2015 and was finished in January 2017. Ethical approval for this study was obtained from Charité Ethical board with the approval ID EA2/122/15. Patient assessment took place at the Charité University Medicine of Berlin, Department of Gynecology with center for oncological surgery. Surgeries have been performed by gynecologic oncology surgeons. All patients signed a written consent to participate in the study. Inclusion criteria were women 18 years or older with a histologically confirmed gynecologic malignancy or with a strong suspicion of a gynecologic malignancy due to imaging and lab results. Borderline tumors were included. The surgery had to be elective and expected to take ≥ 60 min.

The primary endpoint was 30-day postoperative complications classified according to Clavien–Dindo criteria.

### Measures of QoL used in the study

QoL was assessed using the EORTC (European Organization for Research and Treatment of Cancer) Core Quality of Life Questionnaire (QLQ-C30) and the NCCN (National Comprehensive Cancer Network) Distress Thermometer (DT) as well as single-item questions.

The EORTC QLQ-C30 is a second-generation questionnaire designed to measure physical, psychological, and social functioning of patients with cancer. It incorporates nine multi-item scales: five functioning scales (physical, role, cognitive, emotional, and social functioning), three symptom scales (fatigue, pain, and nausea/vomiting), and a global health and QoL scale. The remaining single items assess additional symptoms that are commonly reported by cancer patients: dyspnoea, loss of appetite, sleep disturbance, constipation, and diarrhea and the perceived financial effect of disease and treatment. For the five functional scales and the global QoL scale, a high score represents a good level of functioning. For the symptom scales and items, a high score corresponds to most severe symptoms. It has been validated in multiple studies [8, 11–13]. All scale and item scores were linearly transformed to a scale from 0 to 100.

The NCCN DT is a single-item self-report measure of psychological distress, which consists of a visual analogue tool (resembling a thermometer) with an 11-point scale with the endpoints labeled “No distress”(0) and “Extreme distress” (10), participants were instructed to circle the number that best describes their level of distress within the last week. It was originally developed by Roth et al. to screen the distress of prostate cancer patients. The NCCN added a problem-checklist which contains 37 items divided into 5 groups (practical problems, family problems, emotional problems, physical problems, and spiritual/religious problems) [14]. Patients were asked to choose the items from this list that contributed to their distress within the last week. It has been validated multiple times through comparison with other instruments regarding distress [15, 16].

### Further measurements

Common geriatric assessments performed included the Timed “Up & Go” (TUG)-Test. Statements concerning socio-economic and socio-demographic status, lifestyle, symptoms, mobility and physical functioning were noted, as well as an overall medical and family history, comorbidities and current medication. ASA, ECOG and Karnofsky scores were noted. Questionnaires such as the Charlson Comorbidity Index (CCI), Barthel Index, Instrumental Activities of Daily Living (IADL) Scale, Verbal Numeric Rating Scale (VNRS) for pain, Nutritional-Risk-Screening (NRS), Nursing Delirium Screening Scale (NU-DESC), Mini Mental State Examination (MMSE) and a German questionnaire regarding pelvic floor function (Deutscher Beckenboden-Fragebogen) were carried out.

POC were graded using validated Clavien–Dindo criteria which consist of seven grades (I, II, IIIa, IIIb, IVa, IVb and V). For this project, only severe complications grade IIIb or higher were considered. Grade IIIb is defined as the need of an intervention under general anesthesia. Grade IV is defined as a life-threatening event requiring intensive care unit management and grade V represents a patient’s death [17].

Our assessments were performed 1–3 days preoperatively by medically educated staff and took about 90 min per patient.

Postoperatively each patient was visited daily during rounds and observed for POC. After discharge from the hospital, a follow-up call was made after 3 months to record later occurring POC.

### Statistical analysis

Groups were compared using Fisher’s exact test, Chi test, Kendall´s tau b, Mann–Whitney test or Kruskal–Wallis test. Analysis of the predictive accuracy of continuous variables was assessed by performing Receiver-operator characteristics (ROC) curve analyses to discriminating patients with severe POC from those without them as well as to define cutoffs. Crude and adjusted odds ratios (ORs) with corresponding 95% confidence interval (95% CI) were attained using logistic regression analysis. For the multivariate analysis, gradual logistic regression through all data variables were performed stepwise with *p*_in_ = 0.05 and *p*_out_ = 0.10. Cases with missing data were excluded from the multivariable analyses. *p* < 0.05 was considered statistically significant. IBM® SPSS® Statistics 25 (SPSS Inc. an IBM Company, Chicago, Illinois, USA) was used for statistical analysis.

## Results

Two hundred and thirty seven patients were enrolled in the study. 226 out of 237 (95%) met the intraoperative criteria. 11 patients had to be excluded due to benign pathology or duration of surgery less than 60 min. Included entities were ovarian, fallopian tube, peritoneal cancer (*N* = 155), borderline tumor of the ovary (BOT, *N* = 5), endometrial cancer (*N* = 35), cervical cancer (*N* = 22) and vulvar, vaginal cancer (*N* = 9). The detailed characteristics of our patient collective are shown in Table 1. The results of the measured functional parameters of our patient collective are shown in Table 2.

**Table 1:** Patient characteristics

Characteristics	*N* = 226		
	Clavien–Dindo 0–IIIa *N* = 186	Clavien–Dindo IIIb–V *N* = 40	*p* value
	Median (range)	Median (range)	
Age (years)	59 (18–86)	63 (31–87)	0.3
BMI (kg/m^2^) continous	24.5 (17.5–54.7)	29.12 (20.6–46.4)	< 0.001
	*N* (%)	*N* (%)	
BMI (kg/m^2^) categorical			0.001
Underweight (< 20)	23 (12.4)	0	
Normal (20–25)	100 (53.8)	13 (32.5)	
Overweight (> 25–30)	21 (11.3)	9 (22.5)	
Obese (> 30)	42 (22.6)	18 (45)	
Entities			0.3
Ovarian, fallopian tube, peritoneal cancer	126 (67.7)	29 (72.5)	
Borderline tumor of the ovary	5 (2.7)	0	
Endometrial cancer	30 (16.1)	5 (12.5)	
Cervical cancer	16 (8.6)	6 (15)	
Vulvar, vaginal cancer	9 (4.8)	0

**Table 2:** Functional parameters

Functional parameter	*N* = 226		
	Clavien–Dindo 0–IIIa *N* = 186	Clavien–Dindo IIIb–V *N* = 40	*p* value
	*N* (%)	*N* (%)	
ASA ≥ 3	48 (25.8)	22 (56.4)	< 0.001
ECOG 2–3	9 (4.8)	10 (25)	< 0.001
Polypharmacy (Amount of co-drugs ≥ 5)	29 (15.6)	15 (37.5)	0.003
Barthel Index < 100	15 (8.1)	12 (30)	< 0.001
IADL < 8	7 (3.8)	7 (17.5)	0.004
Charlson Comorbidity Index > 2	47 (25.3)	21 (52.5)	0.001
MMSE < 27	4 (2.2)	3 (7.5)	0.11
TUG > 9.5 s	36 (19.4)	19 (47.5)	< 0.001

*ASA* American Society of Anesthesiologists physical status classification system, *ECOG* Eastern Cooperative Oncology Group performance status, *IADL* Instrumental Activities of Daily Living Scale, *MMSE* Mini Mental State Examination, *TUG* Timed “Up & Go”

40 patients (17.7%) developed a complication grade ≥ IIIb after Clavien–Dindo within 30 days of surgery of whom 9 patients (3.8%) died. The dominant complication was anastomosis insufficiency with 13 patients (5.8%) followed by wound infection (3.5%), sepsis (3.1%) and peritonitis (2.2%). Further types of postoperative complications ≥ IIIb that occurred are shown in Table 3.

**Table 3:** Types and frequency of POC ≥ grade IIIb according to Clavien–Dindo

Type	*N* = 226	
	*N*	%
Anastomosis insufficiency	13	5.8
Wound infection/dehiscence	8	3.5
Pulmonary embolism	7	3.1
Sepsis	7	3.1
Peritonitis	5	2.2
Intestinal perforation	4	1.8
Acute renal failure	4	1.8
CNS complication	4	1.8
Bowel obstruction	3	1.3
Pneumonia	1	0.4
Bilary leak	1	0.4
Secondary haemorrhage	1	0.4

Out of the 40 patients that developed a complication grade IIIb or higher, 29 Patients had ovarian, fallopian tube or peritoneal cancer, representing 72.5% of patients in this group. The remaining 11 patients were made up of 5 patients with endometrial cancer (13%) and 6 patients with cervical cancer (15%). None of the patients with BOT or vulvar, vaginal cancer developed a complication grade IIIb or higher.

### EORTC QLQ-C30

#### Functioning scales

Analysis of the physical functioning scale showed that patients with a score lower than 70 (of 100 total) were more (9 times) likely to develop POC (*p* < 0.001, 95% CI 3.92–21.48) compared to patients with a higher score. Table 4 depicts associations of domains of QoL with POC.

**Table 4:** Results univariate analysis

Characteristics	Unadjusted OR (95% CI)	*p*
Overall condition		
ASA > 2	3.72 (1.82–7.59)	< 0.001
ECOG > 1	10.81 (3.62–32.26)	< 0.001
TUG-Test > 9.5 sek	8.18 (2.58– 25.93)	< 0.001
Barthel Index < 100	4.89 (2.07–11.52)	< 0.001
IADL < 8	5.36 (1.77– 16.3)	0.003
Polypharmacy ≥ 5 co-drugs	3.25 (1.53–6.9)	0.002
CCI > 2	3.27 (1.62–6.6)	0.001
BMI		
> 25 – < 30	6.06 (2.22–16.52)	< 0.001
≥ 30	7.14 (2.65–19.26)	< 0.001
EORTC		
Physical functioning < 70	9.18 (3.92–21.48)	< 0.001
Role functioning ≤ 50	3.29 (1.61–6.73)	0.001
Social functioning < 70	2.15 (1.06–4.35)	0.03
Cognitive functioning < 100	1.81 (0.91–3.61)	0.09
Global QoL ≤ 50	3.34 (1.63–6.85)	0.001
Fatigue > 60	5.19 (2.47–10.92)	< 0.001
Nausea/vomiting > 20	4.77 (2.02–11.26)	< 0.001
Pain > 20	3.18 (1.52–6.65)	0.002
Constipation > 30	3.04 (1.44–6.44)	0.004
Loss of appetite > 50	2.37 (1.08–5.22)	0.03
Sleep disturbances > 50	1.90 (0.94–3.84)	0.07
NCCN DT		
Problems > 10	4.48 (2.19–9.16)	< 0.001
Distress score > 5	2.63 (1.27–5.43)	0.01
Symptoms		
Obstipation	3.63 (1.67–7.92)	0.001
Nausea/vomiting	3.47 (1.6–7.52)	0.002
Bowel obstruction	7.50 (2.44–23.06)	< 0.001
VNRS > 2	1.99 (0.99–3.99)	0.06
German pelvic questionnaire		
Impaired bowel function > 1	3.37 (1.51–7.5)	0.003
Impaired bladder function > 3.5	2.56 (1.23–5.31)	0.01
Sexual function	0.91 (0.4–0.59)	0.5
MMSE < 27	3.67 (0.79–17.08)	0.10

*ASA* American Society of Anesthesiologists physical status classification system, *ECOG* Eastern Cooperative Oncology Group performance status, *TUG-Test* Timed "Up and Go"-Test, *IADL* Instrumental Activities of Daily Living Scale, *CCI* Charlson Comorbidity Index, *BMI* Body Mass Index, *EORTC* European Organisation for Research and Treatment of Cancer Core Quality of Life Questionnaire, *NCCN-DT* National Comprehensive Cancer Network Distress Thermometer, *VNRS* Verbal Numeric Rating Scale for pan, *MMSE* Mini Mental State Examination

#### Symptom scales

Patients who experienced a higher amount of pain were more likely to develop POC. If pain scores were higher than 20 points, the risk was 3 times higher (*p* = 0.002, 95% CI 1.52–6.65) compared to patients with a lower score. Similar results were seen for nausea/vomiting: more than 20 points in this category meant a 5 times higher risk (*p* < 0.001, 95% CI 2.02–11.26) compared to patients with fewer points on this scale. Table 4 depicts associations of domains of QoL with POC.

### NCCN distress thermometer

Analyzing the NCCN distress thermometer showed that higher levels of distress, defined as a score > 5 on the DT, increased the risk of POC by factor 3 (*p* = 0.009, 95% CI 1.27–5.43). Regarding the problems-checklist, our analysis showed that if more than ten items were marked on the list of perceived incriminating problems patients were 5 times more likely to develop POC (*p* < 0.001, 95% CI 2.19–9.16). Table 4 depicts associations of the NCCN Distress Thermometer with POC.

### Further measurements

Data showed that a limited physical status defined as an ASA score above 2 was associated with a higher risk of POC (OR 3.7, 95% CI 1.82–7.59, *p* < 0.001,) as was an ECOG performance status higher than grade 1 (OR 10.8, 95% CI 3.62–32.26, *p* < 0.001). A required time > 9.5 s on the TUG-Test was associated with 8 times higher risk of developing POC (95% CI 2.58–25.93, *p* < 0.001). Evaluation of the Body Mass Index (BMI) also showed significant correlations: being overweight (BMI > 25–30) increased the risk for POC by factor 6 (95% CI 2.22–16.52, *p* < 0.001), being obese (BMI > 30) even increased the risk by factor 7 (95% CI 2.65–19.26, *p* < 0.001) compared to patients with a BMI within the normal range. Results of the multivariate analysis are shown in Table 5.

**Table 5:** Results of multivariate analysis, gradual logistic regression through all data

Characteristics	Adjusted OR (95% CI)	*p*
BMI > 25	5.44 (2.04–14.49)	0.001
EORTC nausea/vomiting > 20	3.08 (1.15–8.26)	0.03
EORTC physical functioning < 70	5.08 (2.23–11.59)	< 0.001
MMSE < 27	7.94 (1.36–45.46)	0.02

*BMI* Body Mass Index, *EORTC* European Organisation for Research and Treatment of Cancer Core Quality of Life Questionnaire, *MMSE* Mini Mental State Examination

## Discussion

Analysis of the collected data showed that some domains of QoL are associated with a considerably higher risk for developing severe postoperative complications. Especially low physical functioning was associated with severe complications following surgery, increasing the risk by factor 5 and making this domain particularly promising for risk assessment.

Regarding symptoms, we especially found associations between postoperative complications and nausea/vomiting, increasing the risk by factor 3. This could be due to our study population that consisted mainly of women with ovarian cancer which frequently leads to ascites and/or acute/subacute bowel obstruction and could, therefore, cause an increase of these symptoms. Quinten et al. correspondingly found that the aspects of QoL that have a prognostic value depend on the cancer type and in ovarian cancer nausea/vomiting was a prognostic factor for survival [28].

Regarding the patients’ BMI being overweight or obese meant a 5 times higher risk for adverse outcome. A high BMI has been found to influence physical, functional and social well-being negatively and obese patients appear to have a lower QoL than normal-weight patients with gynecologic malignancies [29, 30]. A high BMI can also be an impairment for wound healing and/or mobility that may lead to adverse events independently from the patient’s QoL.

In our study, a Mini Mental State Examination (MMSE) < 27 showed a possible risk increase by factor 8 for severe postoperative complications. Schmidt et al. did find impaired self-reported cognitive function in the EORTC as well as low MMSE results predictive for overall mortality in onco-surgical patients [7]. Several studies also directly point out cognitive impairment to be a risk factor for POC, but most of them only included geriatric patients and mainly referred to gastrointestinal cancer surgery [31, 32]. Cognitive impairment and its effect on POC have not been examined in gynecological oncology so far and appear to be a promising subject for further research.

Most studies that assess preoperative QoL have different endpoints: baseline QoL has been found to be predictive of overall survival, progression-free survival or non-surgical complications in cancer patients [4–6, 18–20]. In gynecologic cancer patients, QoL was found to be prognostic for overall survival, progression-free survival and chemotherapy toxicity [21–24]. Roncolato et al. found that low baseline global health status, role function and physical function before chemotherapy were associated with early termination of chemotherapy in patients with ovarian cancer [25].

To our knowledge, there are currently just two studies available investigating the effect of QoL on surgical outcome in gynecological cancer patients: Doll et al. and Baker et al. found that lower preoperatively assessed QoL scores—especially low physical and functional scores—were associated with an increased risk of POC and 30-day readmission [26, 27]. This fits our results that imply the physical functioning scale to be the most significant parameter.

The strengths of our investigation are its prospective design as well as the use of detailed geriatric assessment tools. Daily visits of each patient after surgery have contributed to the internal validity and reliability of our data.

It is assumed that the evaluation of QoL is complex for the clinical work. However, preoperative evaluation of QoL using only one questionnaire like the European Organisation for Research and Treatment of Cancer Quality of Life questionnaire QLQ-C30 questionnaire can help the surgeon to identify the high-risk patients for severe postoperative complications.

Our study has several limitations. Patients presented with different types of malignancies in different stages. The patient collective is made up mainly of patients with ovarian cancer. The surgical approach to treat ovarian cancer is clearly very different from the approach to treat, for example, vulvar cancer. For that reason, it is important to note that no patients with BOT or vulvar, vaginal cancer were part of the analyzed group of patients with POC ≥ IIIb. Therefore, all patients in the group with POC ≥ IIIb had major abdominal surgery, which improves the comparability in this group. Still, this is clearly a limitation of our study.

## Conclusion

Preoperative measurement of quality of life can predict postoperative complications in gynecological cancer surgery. Specific domains of QoL may provide prognostic information and measuring them preoperatively could help to predict severe postoperative complications in patients with gynecological cancer. Based on our study, preoperative assessment of QoL should be routinely established for patients undergoing gynecological cancer surgeries. Our data demonstrate that symptoms are relevant for risk assessment. Future studies should investigate whether preoperative symptom control can reduce the rate of postoperative complication and underline the need for prehabilitation approaches.

## Data Availability

All data are available as SPPS file.
