# Correction to: Preoperative quality of life as prediction for severe postoperative complications in gynecological cancer surgery: results of a prospective study

**DOI:** 10.1007/s00404-021-06333-y

**Published:** 2021-11-19

**Authors:** Jalid Sehouli, Kathrin Heise, Rolf Richter, Hannah Woopen, Louise Anders, Melisa Guelhan Inci

**Affiliations:** Department of Gynecology with Center of Surgical Oncology, Charité University Hospital of Berlin, Augustenburger Platz 1, 13353 Berlin, Germany

## Correction to: Archives of Gynecology and Obstetrics (2021) 303:1057–1063 10.1007/s00404-020-05847-1

The article “Preoperative quality of life as prediction for severe postoperative complications in gynecological cancer surgery: results of a prospective study” written by Jalid Sehouli, Kathrin Heise, Rolf Richter, Hannah Woopen, Louise Anders and Melisa Guelhan Inci was originally published electronically on the publisher’s internet portal on October 29, 2020 without open access. With the author(s)’ decision to opt for Open Choice the copyright of the article changed to © The Author(s) 2020 and the article is forthwith distributed under a Creative Commons Attribution 4.0 International License, which permits use, sharing, adaptation, distribution and reproduction in any medium or format, as long as you give appropriate credit to the original author(s) and the source, provide a link to the Creative Commons licence, and indicate if changes were made. The images or other third-party material in this article are included in the article’s Creative Commons licence, unless indicated otherwise in a credit line to the material. If material is not included in the article’s Creative Commons licence and your intended use is not permitted by statutory regulation or exceeds the permitted use, you will need to obtain permission directly from the copyright holder. To view a copy of this licence, visit http://creativecommons.org/licenses/by/4.0/. Open Access funding enabled and organized by Projekt DEAL.

The original article has been updated.

